# Pitfalls in Comparing Mechanical Circulatory Support Devices Using Administrative Datasets: Epidemiology of the Diseased Population

**DOI:** 10.1016/j.jscai.2025.103998

**Published:** 2025-11-04

**Authors:** Tayyab Shah, Chantal Holy, Ali Almedhychy, Jeffrey W. Moses, Helen Parise, Alejandro Lemor, William W. O’Neill, Alexandra J. Lansky

**Affiliations:** a Yale Cardiovascular Research Group, Yale School of Medicine, New Haven, Connecticut; b Division of Cardiovascular Medicine, Hospital of the University of Pennsylvania, Philadelphia, Pennsylvania; c Johnson & Johnson, New Brunswick, New Jersey; d Johnson & Johnson, Danvers, Massachusetts; e Division of Cardiology, NewYork-Presbyterian Hospital/Columbia University Irving Medical Center, New York, New York; f Division of Cardiology, St. Francis Hospital & Heart Center, Roslyn, New York; g Division of Cardiology, University of Mississippi Medical Center, Jackson, Mississippi; h Division of Cardiology, Henry Ford Hospital System, Detroit, Michigan

**Keywords:** high-risk percutaneous coronary intervention, impella, intra-aortic balloon pump, patient characteristics, propensity score matching

## Introduction

Both Impella, a percutaneous ventricular assist device (pLVAD), and intra-aortic balloon pump (IABP) provide hemodynamic support for patients undergoing high-risk percutaneous coronary intervention (HRPCI).^1^ In light of limited randomized data directly comparing these devices,^2^ observational studies leveraging administrative health datasets, such as the PREMIER Healthcare Database, have sought to evaluate their relative efficacy.^3^, ^4^, ^5^ This study assesses the limitations of such analyses using a contemporary PREMIER cohort.

## Materials and methods

Patients undergoing their first elective HRPCI (defined as PCI with pLVAD or IABP) between 2018 and April 2024 who received pLVAD (5A0221D/5A0211D) or IABP (5A02210/5A02110) on the same day as the procedure (but not both), did not have cardiogenic shock on admission (R57.0), and did not receive surgery on the index admission were identified in the PREMIER Healthcare Database. We calculated propensity scores using logistic regression of 87 independent preprocedural variables (demographic characteristics, comorbidities, hospital characteristics, and admission/presentation characteristics) present in more than 1% of included patients, with standardized mean differences (SMDs) between the 2 arms exceeding 0.01. Variable-rate propensity score matching without replacement using a caliper width of 0.03 was performed to match patients in the IABP arm to those in the pLVAD arm. In this study, we compared characteristics among the unmatched and matched groups and identify which patients are excluded/discounted after matching. The full details of the methods and results of the propensity matched analysis have been published separately.^6^ SMDs were used to assess differences between cohorts. All analyses were conducted using R version 4.2.2 and RStudio (2024.04.1 Build 748). This study was reviewed by the New England Institutional Review Board and deemed exempt from review.

## Results

**Figure 1:**
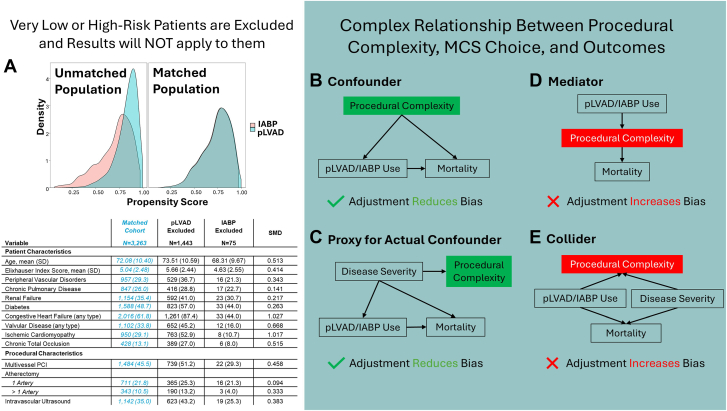
**Pitfalls in interpretation and adjustment of propensity matched analysis comparing pLVAD with IABP in HRPCI.** (**A**) Distribution of propensity scores before and after matching. Directed acyclic graphs are graphs with arrows showing the direction of causal effects between variables.^8^ We present the complex relationship of procedural complexity (eg, multivessel revascularization and atherectomy) with MCS choice and mortality using directed acyclic graphs showing how procedural complexity can be a (**B**) confounder; (**C**) proxy for actual confounder; (**D**) mediator; and (**E**) collider. HRPCI, high-risk percutaneous coronary intervention; IABP, intra-aortic balloon pump; pLVAD, percutaneous left ventricular assist device.

In total, 4781 patients (3859 pLVAD and 922 IABP) met eligibility criteria. Patients in the pLVAD arm were older (73 ± 10.5 vs 71 ± 10.2 years) had higher Elixhauser comorbidity scores (5.3 vs 5.0) and underwent more multivessel revascularization. After generating propensity scores for treatment with pLVAD, there was overlap among patients in the IABP and pLVAD arms for the intermediate scores; however, patients with lower scores almost exclusively received IABP, while those with higher scores almost exclusively received pLVAD (Figure 1). A total of 3363 patients were matched (847 IABP to 416 pLVAD), meaning 1443 were excluded from the pLVAD group, while only 75 were excluded from the IABP group. Compared with patients excluded from the IABP arm, patients excluded from the pLVAD arm were older (74 ± 10.6 vs 68 ± 9.7 years), had higher Elixhauser comorbidity scores (mean, 5.7 vs 4.6), and had more comorbidities including diabetes (57% vs 44%), chronic kidney disease (41% vs 31%), and peripheral vascular disease (37% vs 21%). They also had more complex cardiac disease with substantially higher rates of congestive heart failure (87% vs 44%), valvular disease (45% vs 16%), and prior chronic total occlusions (27% vs 8%). Finally, the excluded patients from the pLVAD group also underwent more multivessel PCI (51% vs 29%) and atherectomy (39% vs 25%; all SMDs > 0.1).

## Discussion and conclusion

This study highlights limitations in comparing outcomes among patients receiving IABP-supported vs pLVAD-supported HRPCI using observational data. Despite only including elective cases and excluding patients with shock, pLVAD recipients had more comorbidities and underwent more complex procedures, reflecting significant baseline differences and inherent selection bias which cannot be completely adjusted for due to the limited data available in administrative datasets. Additionally, propensity score analysis revealed minimal overlap at the extremes, with IABP preferentially used in lower-risk patients and pLVAD used in higher-risk patients. This likely reflects an assumed lack of clinical equipoise between the 2 devices on either risk extreme by interventionalists in aggregate. Because of this, any observational study comparing the 2 devices (including those using other methods such as inverse probability weighting) is inherently limited to assessing intermediate-risk patients and will inevitably exclude or heavily discount the highest risk patients—the very patients presumed to benefit most from pLVAD use by limiting hemodynamic deterioration. Moreover, these limitations exist in addition to the well documented challenges with determining the timing and indication for mechanical circulatory support (MCS) use (ie, prophylactic vs bailout) in administrative datasets.^3^, ^4^, ^5^

Furthermore, procedural characteristics such as multivessel HRPCI or atherectomy use have a more complex relationship with MCS strategy and outcomes than is often appreciated. Adjusting for these procedural variables in the propensity score—as done in many prior analyses—can introduce unintended bias, depending on whether the variables are true confounders, risk modifiers, and/or colliders (definitions in Supplemental Table S1). While adjustment for confounders and/or proxies for unmeasured confounding (such as factors related to disease severity) can reduce bias when estimating the total causal effect of MCS on outcomes, caution is warranted. Specifically, many of these variables also act as risk mediators and colliders and should generally not be adjusted for, as this can bias results and attenuate the estimated total treatment effect of MCS (Figure 1; Supplemental Table S1).^7^^,^^8^ Finally, it is important to also adjust for hospital differences as some sites might have lack of availability or familiarity with certain devices (most likely pLVAD). This can be achieved by adjusting for hospital level clustering in the statistical model and/or excluding patients from sites without prior procedures with both devices. Without adjusting for this would likely bias results to favor pLVAD because sites without pLVAD access may be forced to place IABP in sicker patients in lieu of other support.

Overall, these findings highlight the inherent challenges of observational studies in this setting and the importance of cautious interpretation. In the absence of robust randomized evidence—and given the practical and ethical challenges of randomizing high-risk patients most likely to benefit from pLVAD due to perceived lack of equipoise—observational studies will remain central to clinical decision making. Future registry-based research evaluating MCS-supported HRPCI should address these methodological limitations by first classifying treatment-related differences into true confounders, risk mediators, and colliders and by avoiding adjustment for mediators and colliders that could bias results. Careful selection of covariates is essential to ensure that adjustment reduces rather than amplifies bias, enabling the most accurate possible estimates of treatment effect.
