# Supplementary material for: Pitfalls in Comparing Mechanical Circulatory Support Devices Using Administrative Datasets: Epidemiology of the Diseased Population
Source: J Soc Cardiovasc Angiogr Interv. 2025 Nov 4;4(12):103998. doi: 10.1016/j.jscai.2025.103998 (PMC12766040; doi:10.1016/j.jscai.2025.103998)
Supplement: Supplemental Table S1 [file mmc1.docx]

***Supplemental Table S1.***

| **Variable Type** | **Definition** | **Role in Causal Pathway** | **Example of Procedural Complexity** | **Effect if Adjusted For** | **Adjustment recommendation*** |
| --- | --- | --- | --- | --- | --- |
| **Confounder** | A variable associated with both the exposure and the outcome, but not caused by the exposure. | Common cause of exposure and outcome. | Planned multivessel HRPCI influences MCS choice and subsequent mortality and is a proxy for other confounders (such as coronary artery disease severity) | Removes bias from confounding. | **Yes** – to estimate total effect. |
| **Mediator** | A variable that lies between the exposure and outcome, carrying part of the causal effect. | On the direct causal pathway from exposure to outcome. | pLVAD support enables more complete multivessel revascularization [9] which could impact post-HRPCI mortality | Removes part of the causal effect (estimates only *direct* effect). | **No** – unless goal is to estimate direct (not total) effect. |
| **Collider** | A variable that is influenced by both the exposure and the outcome (or their causes). | Common *effect* of exposure and outcome (or their causes). | Procedural complexity is influenced by both baseline disease severity (a cause of mortality) and pLVAD use | Introduces spurious associations (collider bias). | **No** – avoid conditioning or adjusting. |

*Assumes the goal is to estimate the **total causal effect** of the exposure on the outcome
